# Comparative transcriptomics between species attributes reactogenicity pathways induced by the capsular group B meningococcal vaccine, 4CMenB, to the membrane-bound endotoxin of its outer membrane vesicle component

**DOI:** 10.1038/s41598-019-50310-0

**Published:** 2019-09-24

**Authors:** Dylan Sheerin, Daniel O’Connor, Christina Dold, Elizabeth Clutterbuck, Moustafa Attar, Christine S. Rollier, Manish Sadarangani, Andrew J. Pollard

**Affiliations:** 1 Oxford Vaccine Group, Department of Paediatrics, University of Oxford, and the NIHR Oxford Biomedical Research Centre, Centre for Clinical Vaccinology and Tropical Medicine, Churchill Hospital, Oxford, UK; 2 Wellcome Centre for Human Genetics, University of Oxford, Oxford, UK; 3 Vaccine Evaluation Center, BC Children’s Hospital Research Institute, Vancouver, BC Canada; 4 Department of Pediatrics, University of British Columbia, Vancouver, BC Canada

**Keywords:** Genetics research, Vaccines, Paediatric research

## Abstract

The capsular group B meningococcal (MenB) four component vaccine (4CMenB) has been licensed for the prevention of invasive disease caused by MenB. The vaccine causes fever in infants, particularly when given in combination (concomitant) with other routinely-administered vaccines (routine), such as the standard diphtheria, tetanus, pertussis (DTP)-containing vaccine. To assess the suitability of a mouse immunisation model to study this phenomenon, we monitored temperature in mice after a second dose of routine vaccines, with or without 4CMenB, and compared the results with those in humans. Using this mouse model, we explored the reactogenicity of 4CMenB components by measuring changes in temperature, cytokines, and gene expression induced by 4CMenB, one of its components, wild-type or attenuated endotoxin outer membrane vesicles (OMVs), or lipopolysaccharide (LPS). A significant rise (*p* < 0.01) in temperature was observed in mice immunised with 4CMenB, wild-type OMVs, and LPS. RNA-sequencing of mouse whole blood revealed a gene signature shared by the 4CMenB, OMV, and LPS groups consisting of bacterial pattern recognition receptors and neutrophil activation marker genes. Sequencing of neutrophils isolated after concomitant 4CMenB identified cells expressing the OMV-associated genes *Plek* and *Lcp1*. Immunisation with 4CMenB or OMVs led to increased IL-6 in serum and significant upregulation (*p* < 0.0001) of prostaglandin-synthesising enzymes on brain tissue. These data demonstrate the suitability of a mouse model for assessing vaccine reactogenicity and strongly indicate that the fever following vaccination with 4CMenB in human infants is induced by endotoxin contained in the OMV component of the vaccine.

## Introduction

The Gram-negative bacterium *Neisseria meningitidis* (*N*. *meningitidis*) is the causative agent of invasive meningococcal disease (IMD), a fulminant bacterial infection with the highest incidence in the first year of life^1,2^. IMD can cause life-threatening sepsis and meningitis and it is the leading cause of infectious death in UK infants; a proportion of survivors are left with long-term sequelae including cognitive impairment, hearing loss, chronic pain, scarring and loss of limb^3^. Meningococcal capsular group B (MenB) has become the leading cause of IMD in the UK, responsible for 80–90% of cases^4^. A four component MenB vaccine (4CMenB) was developed and licensed for use in several countries with the aim of preventing IMD caused by this capsular group^5^. The UK was the first country to include this vaccine in the routine immunisation schedule in 2015 and it is given in three doses at 2, 4, and 12 months of age^6^.

Early post-implementation surveillance in the UK indicated that 4CMenB was effective at preventing invasive disease caused by MenB, with an effectiveness of 83% resulting in a halving of all cases in the vaccine-eligible group within 10 months of the introduction of the two-dose schedule^7^. However, concerns remain about the reactogenicity associated with the vaccine, which is exacerbated when 4CMenB is administered concomitantly with vaccines such as the standard diphtheria, tetanus, pertussis (DTP)-containing vaccine, routinely-administered to infants in the UK; fever rates (≥38.5 °C) in the 4CMenB + routine immunisation cohort were nearly double (50–60%) those observed in infants receiving routine immunisations alone in phase III trials^8^. The outer membrane vesicle (OMV) component of the vaccine contributes to the reactogenicity of the vaccine; early studies of 4CMenB recombinant proteins – the factor H binding protein (fHbp), neisserial heparin-binding antigen (NHBA), and neisserial adhesin A (NadA) – in infants found a slight increase in local and systemic reactogenicity associated with the inclusion of OMVs^9,10^. This is likely due to quantities of membrane-bound endotoxin found in OMV preparations, although OMVs prepared by deoxycholate extraction (dOMVs), such as those in 4CMenB and other OMV-based vaccines, have reduced proportions of endotoxin compared with untreated native OMVs (nOMVs)^11^.

Paracetamol prophylaxis was found to significantly decrease fever after concomitant 4CMenB immunisation without negatively impacting on the immunogenicity^12^, and Public Health England (PHE) have since made the recommendation that infants in the UK be given paracetamol after receiving the first and second dose of the vaccine^13^. While post-immunisation fever is typically a benign event, with mild to moderate and transient manifestations^14^, it represents a challenge for clinicians who are faced with the difficulty of differentiating adverse events following immunisation (AEFI) from significant intercurrent bacterial infection in infants^15^. Data are beginning to emerge on the healthcare burden of 4CMenB-related AEFI and highlight a small but significant increase in emergency department attendances in the region of 2–3%^16–20^. Under the current guidelines of the National Institute for Health and Care Excellence (NICE), presentation with AEFI in those under 3 months of age may result in unnecessary hospitalisation, invasive diagnostic procedures such as lumbar punctures, or the administration of antibiotics^21^. Therefore, it is of clinical importance to understand the aetiology of post-immunisation fever.

In order to elucidate the host molecular pathways associated with the immunogenicity and reactogenicity of 4CMenB and other OMV vaccines, a European Union Childhood Life-threatening Infectious Disease Study (EUCLIDS) was conducted by the Oxford Vaccine Group (OVG), University of Oxford. The clinical study focused on investigating changes in gene expression in infants after their four month dose of vaccines routinely-administered to UK infants (at the time of study), with or without concomitant 4CMenB^22^. Post-immunisation whole blood gene signatures were characterised and correlated with temperature and immunogenicity data (GSE131929). To further explore the reactogenicity of concomitant 4CMenB immunisation, and to determine the relative contribution of each of its components to these reactions, we sought to assess temperature and gene expression changes in groups of mice immunised with 4CMenB, on its own or with routine vaccines, or one of its four alum-adsorbed components. The role of OMVs and membrane-bound endotoxin in driving this reactogenicity was also examined by comparing the 4CMenB NZ98/254 dOMVs with those of another strain, H44/76, and with nOMVs from this strain genetically-engineered to express an attenuated form of endotoxin (lpxL1). These data provide novel insights into 4CMenB reactogenicity through comparative analyses of its components.

## Results

### 4CMenB immunisation increases temperature in mice when administered on its own or concomitantly with routine immunisations

**Figure 1:**
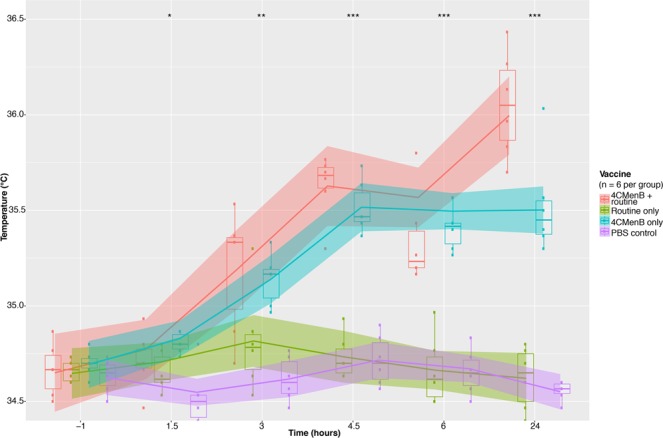
Changes in mouse surface temperature associated with vaccine combinations, 24 hours after the second dose of vaccine. Overall trend in temperature from baseline to 24 hours after the second doses of each three-vaccine combination for each group. Loess smoothing was applied to group temperature trend lines. Group medians were compared between vaccine groups at each time point using a Kruskal-Wallis test. *< 0.05, **< 0.01, ***< 0.001, ****< 0.0001.

Mice were immunised with 1/15 of the human dose of each of four three-vaccine combinations: 4CMenB plus the 5-in-1 diphtheria, tetanus, acellular pertussis, inactivated polio, *Haemophilus influenzae* type B vaccine (DTaP-IPV-Hib) and the 13-valent pneumococcal conjugate vaccine (PCV13 (4CMenB + routine group), DTaP-IPV-Hib with PCV13 and phosphate-buffered saline (PBS) to control for a third immunisation (routine only group), three doses of PBS (PBS control group), or 4CMenB with two doses of PBS – at day zero and day 21 (Supplementary Fig. 1). Non-contact infrared thermometry was used to measure the surface temperature of mice at baseline on day 21 (60 minutes before second dose), and every 90 minutes after the second dose up to six hours, and 24 hours after the second dose (Fig. 1). A statistically significant rise (*p* < 0.05) in temperature occurred as early as three hours after the second dose in the 4CMenB and 4CMenB + routine groups (Fig. 1), continuing to rise up to 24 hours after this dose (*p* < 0.01) with a higher temperature observed in the 4CMenB + concomitant group at 24 hours (*p* = 0.007576). No such rise in temperature was observed for the routine only or PBS control groups (Fig. 1). This finding demonstrates an increased incidence of fever associated with concomitant 4CMenB in the mouse immunisation model, recapitulating what is observed in human studies.

**Figure 2:**
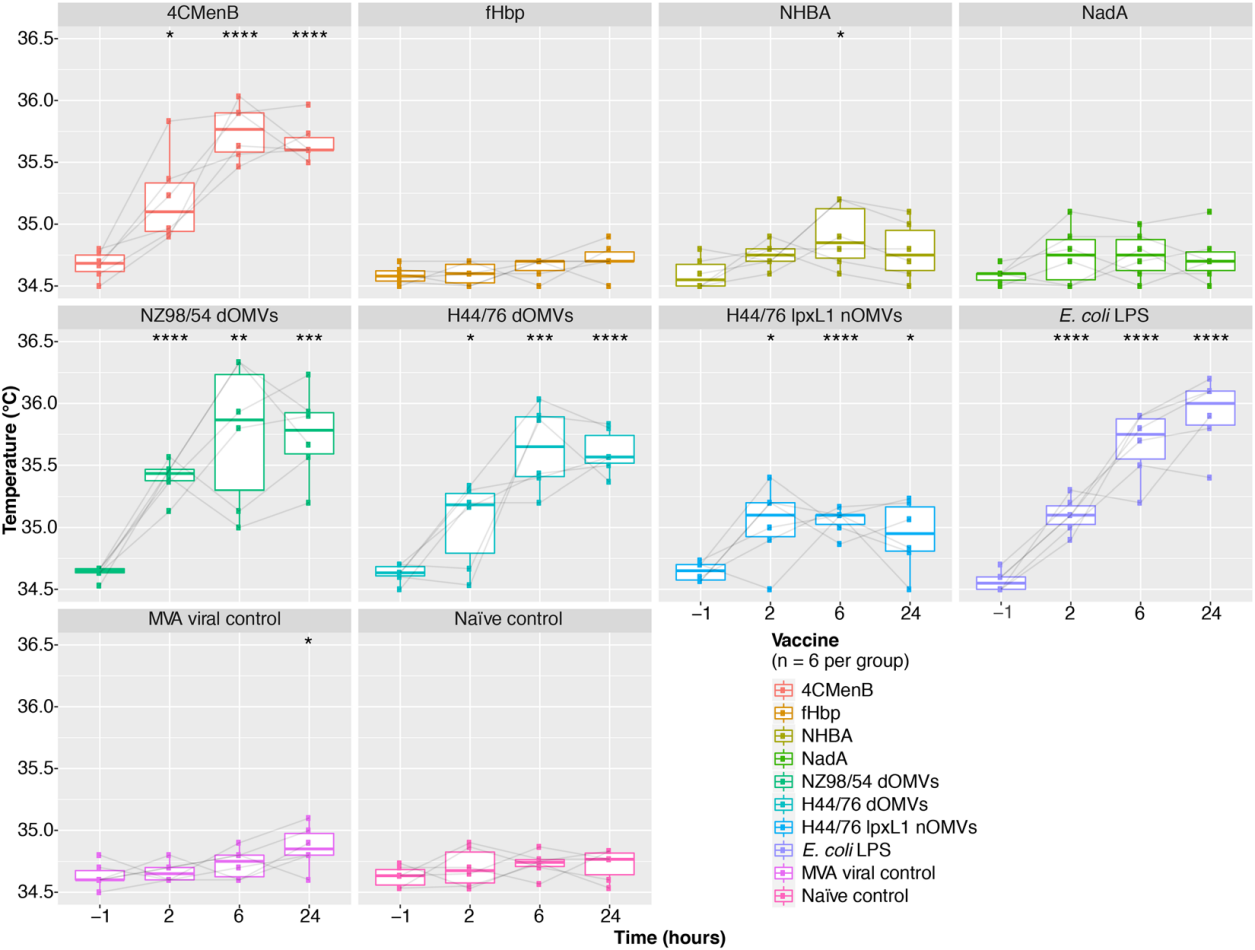
Changes in mouse surface temperature associated with 4CMenB, its individual components, and comparator immunisations, 24 hours after the second dose of vaccine. Boxplots depicting the change in temperature from baseline to 24 hours after the second dose of vaccine/component for each individual group. Vaccine group medians at each timepoint were compared with baseline for that vaccine using a Wilcoxon signed-rank test. *< 0.05, **< 0.01, ***< 0.001, ****< 0.0001.

Groups of mice were immunised with 1/5 of the human dose of 4CMenB, each of the four components of 4CMenB administered individually with alum (Table 1), dOMVs from the H44/76 strain, lpxL1 nOMVs from the same strain, *Escherichia coli* (*E*. *coli*) lipopolysaccharide (LPS) in alum, and modified vaccinia Ankara (MVA) – at day/zero and 21 (Supplementary Fig. 2). The surface temperature of each mouse was measured to determine changes in temperature associated with the second dose of each of these vaccines/components. Temperature readings were taken at baseline (60 minutes before second dose), two, six, and 24 hours after the second dose (see Fig. 2). Only mice that received OMV vaccines, specifically those containing dOMVs, displayed a statistically significant rise in temperature relative to the naïve control group. All OMVs induced a statistically significant increase in temperature relative to pre-immunisation, but a greater rise in temperature was observed for the dOMV groups than the lpxL1 nOMV group, particularly at 24 hours post-immunisation. This demonstrates that attenuation of membrane-bound endotoxin reduces fever in these mice.

**Table 1:** Composition of the capsular group B meningococcal four component vaccine, 4CMenB.

Vaccine constituent	Quantity
Neisserial adhesin A (NadA) variant 3.1	50 μg
Factor H binding protein (fHbp) variant 1.1 (fused to GNA2091)	50 μg
Neisserial heparin binding antigen peptide ID 2 (fused to GNA1030)	50 μg
Outer membrane vesicles from the NZ98/254 strain	25 μg
Aluminium hydroxide	0.5 mg
Sodium chloride, histidine, sucrose, and water for injections	—

The final formulation of the 4CMenB vaccine as outlined by the European Medicines Agency^59^.

### Mouse transcriptional responses to 4CMenB immunisation correlate with those of human infants 24 hours after concomitant 4CMenB

**Figure 3:**
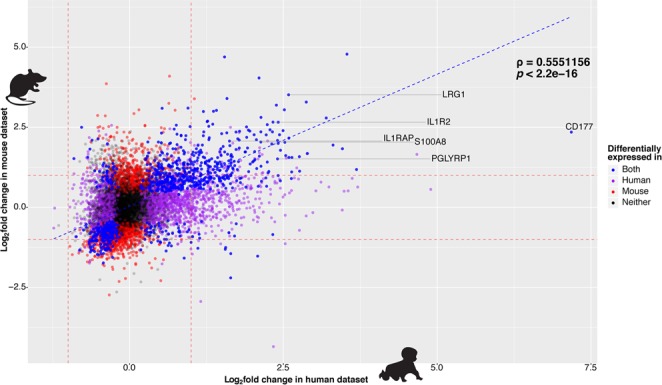
Agreement between mouse and infant datasets. Agreement plot of log_2_ fold changes (LFCs) associated with all genes with one-to-one orthologs from the mouse 4CMenB group (y-axis) and the infant 4CMenB + routine immunisations group (x-axis) at 24 hours after the second dose. Spearman correlation rho value calculated between genes found to be significantly differentially expressed (FDR-adjusted *p*-value < 0.01) in both species (highlighted in blue) is shown beside the dotted blue trend line, with the significance of the correlation indicated by the *p*-value below. The remaining genes are coloured purple, red, or black if they were significantly differentially expressed only in humans, mice, or neither, respectively. Several genes of interest based on the downstream analysis in the infant study are labelled and connected to their corresponding point.

RNA-sequencing (RNA-seq) was performed on RNA extracted from mouse whole blood 24 hours after the second dose of immunisation with 4CMenB or one of the comparator test groups. To determine whether changes in the mouse transcriptome associated with 4CMenB immunisation were comparable with those seen at the same time point in the concomitant group of the infant study, significant genes (false discovery rate (FDR)-adjusted *p*-value < 0.01) were ranked by log_2_ fold change (LFC) relative to the naïve control group and converted to human orthologs where possible. Spearman rank correlation indicated moderate correlation (*ρ* = ~0.55) between one-to-one orthologs, but the association was highly significant (*p* < 2.2^−16^). An agreement plot of differentially expressed orthologs from each genus 24 hours after second dose of immunisation is shown in Fig. 3, with common significantly differentially expressed genes (DEGs) highlighted in blue.

### Mouse whole blood gene signatures cluster by vaccine type

**Figure 4:**
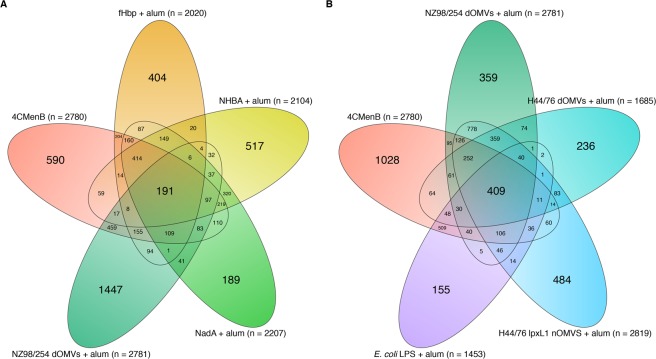
Global changes in gene expression at 24 hours. Euler plots of the total number of significantly differentially expressed genes (DEGs, FDR-adjusted *p*-value < 0.01) associated with each test group 24 hours after the second dose. (**A**) Overlap between DEGs associated with 4CMenB, and each of its alum-adsorbed components. (**B**) Overlap between 4CMenB, each of the alum-adsorbed deoxycholate and native outer membrane vesicles (dOMVs and nOMVs), and alum-adsorbed lipopolysaccharide (LPS) from *Escherichia coli*.

Initial assessment of the global changes in gene expression associated with 4CMenB, 4CMenB components, and comparator test groups identified a large number of DEGs associated with each group (Fig. 4). NZ98/254 OMV immunisation was associated with the greatest number of significantly (FDR-adjusted *p*-value < 0.01) DEGs, and the component of 4CMenB with the greatest number of uniquely DEGs at 24 hours after the second dose (Fig. 4A). A greater overlap between significant DEGs was observed between 4CMenB, OMV, and *E*. *coli* LPS groups (Fig. 4B) than was observed for 4CMenB and its constituent antigens. Of the significantly DEGs associated with 4CMenB and its components, only 191 DEGs were common to the four components and the final formulation, whereas 409 DEGs were common to 4CMenB, the three OMV groups, and *E*. *coli* LPS.

**Figure 5:**
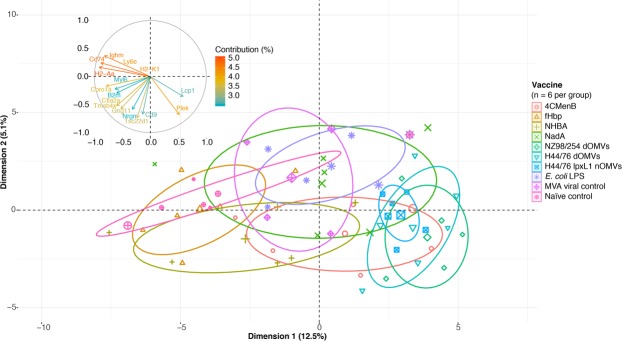
Early gene signatures cluster according to vaccine type. Two-dimensional representation of principal components (PCs) one and two as determined by PC analysis of gene expression values from all groups. Ellipses correspond to 95% confidence intervals for each group. The contribution plot in the upper left quadrant depicts the percentage contribution of individual genes to the clustering observed along each dimension.

Using principal component (PC) analysis (PCA), the data clustered by vaccine type, with the three OMV groups and three protein groups forming distinct clusters on the first two PCs accounting for 12.5% and 5.1% of the variation, respectively (Fig. 5). The majority of this clustering was driven by a small group of genes, with the histocompatibility antigen genes, *H2-Aa*, *H2-K1* and *Cd74*, the lymphocyte antigen gene *Ly6e*, and the immunoglobulin *Ighm* gene contributing most to the clustering (Fig. 5). This suggests that antigen-specific processing and presentation pathway genes account for the greatest divergence in early gene response signatures between the groups tested. The genes encoding a protein involved in lymphocyte interaction, *Lcp1*, and a degranulation protein, *Plek*, contribute most to the clustering of the three OMVs groups.

### Gene signatures induced by 4CMenB and OMVs are defined by bacterial innate response genes

**Figure 6:**
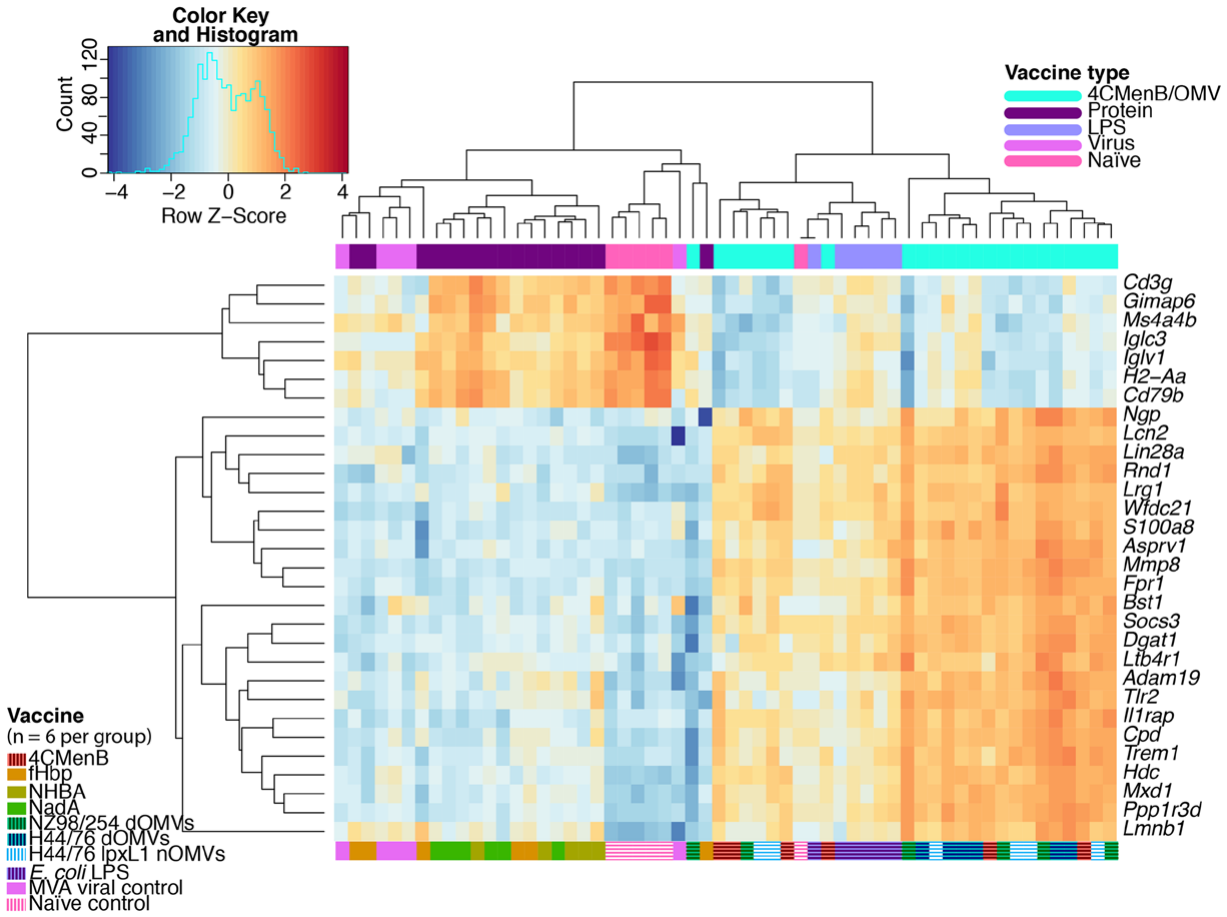
Top 30 most variable genes across all samples. Pairwise Euclidian distances between all samples were calculated from the normalised counts of genes with the maximum dispersion from the population mean and are represented by the column and row dendrograms. Heatmap tiles correspond to row z scores, centred and scaled upon the mean expression value for that gene across the dataset, with orange and blue tiles indicating increased or decreased expression relative to the population mean, respectively.

Unsupervised hierarchical clustering of the most variable genes across all samples determined two divergent clusters defined by the recombinant protein and viral control groups on the one hand and the 4CMenB and the OMV groups on the other (Fig. 6). The classifying genes were broadly related to innate immune responses, such as pattern recognition receptor (PRR), antigen processing and presentation, and cell signalling pathway genes. Approximately one third of these genes were related to neutrophils – *Ngp*, *Lcn2*, *Lrg1*, *S100a8*, *Mmp8*, *Fpr1*, *and Ltb4r* – all of which have roles in neutrophil migration, anti-bacterial responses, and differentiation. Several of the classifier genes found to have decreased expression in the 4CMenB and OMV groups relative to the recombinant protein, MVA, and naïve control groups were associated with antigen recognition and presentation, as was observed by PCA. These included the T cell receptor gene *Cd3g*, the immunoglobulin lambda constant and variable chain genes *Iglc3* and *Iglv1*, the major histocompatibility complex gene *H2-Aa*, and the B cell receptor gene *CD79b* (Fig. 6). Most of these genes had moderate to low expression in the MVA and fHbp groups but were highly expressed in the NHBA and NadA groups, suggesting a similar immune response to these two recombinant proteins.

**Figure 7:**
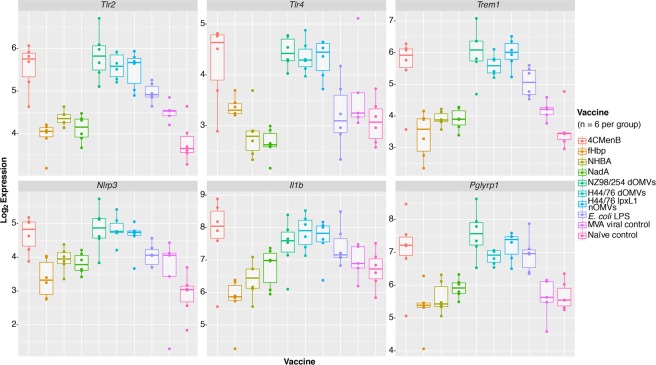
Selection of bacterial pattern recognition receptor and downstream effector genes. Normalised log_2_ expression values for genes encoding several PRRs and downstream innate immune responses, among the top genes identified as significantly differentially regulated across all samples.

A further examination of a selection of PRRs highlighted a trend in the expression of toll-like receptors (TLRs) specific for bacterial pathogen-associated molecular patterns (PAMPs), particularly*Tlr2* and *Tlr4*, as significantly differentially expressed (DE) in the 4CMenB and OMV groups (Fig. 7 and Supplementary Fig. 3). A further examination of bacterial PAMP receptors and downstream effectors showed a similar pattern of differential expression (DE) associated with genes encoding a peptidoglycan receptor, *Pglyrp1*, a TLR4-amplifying receptor, *Trem1*, an inflammasome activator, *Nlrp3*, and the cytokine 1L-1β associated with these groups (Fig. 7 and Supplementary Fig. 4).

### Concomitant 4CMenB immunisation stimulates proinflammatory gene expression in a cluster of activated neutrophils

**Figure 8:**
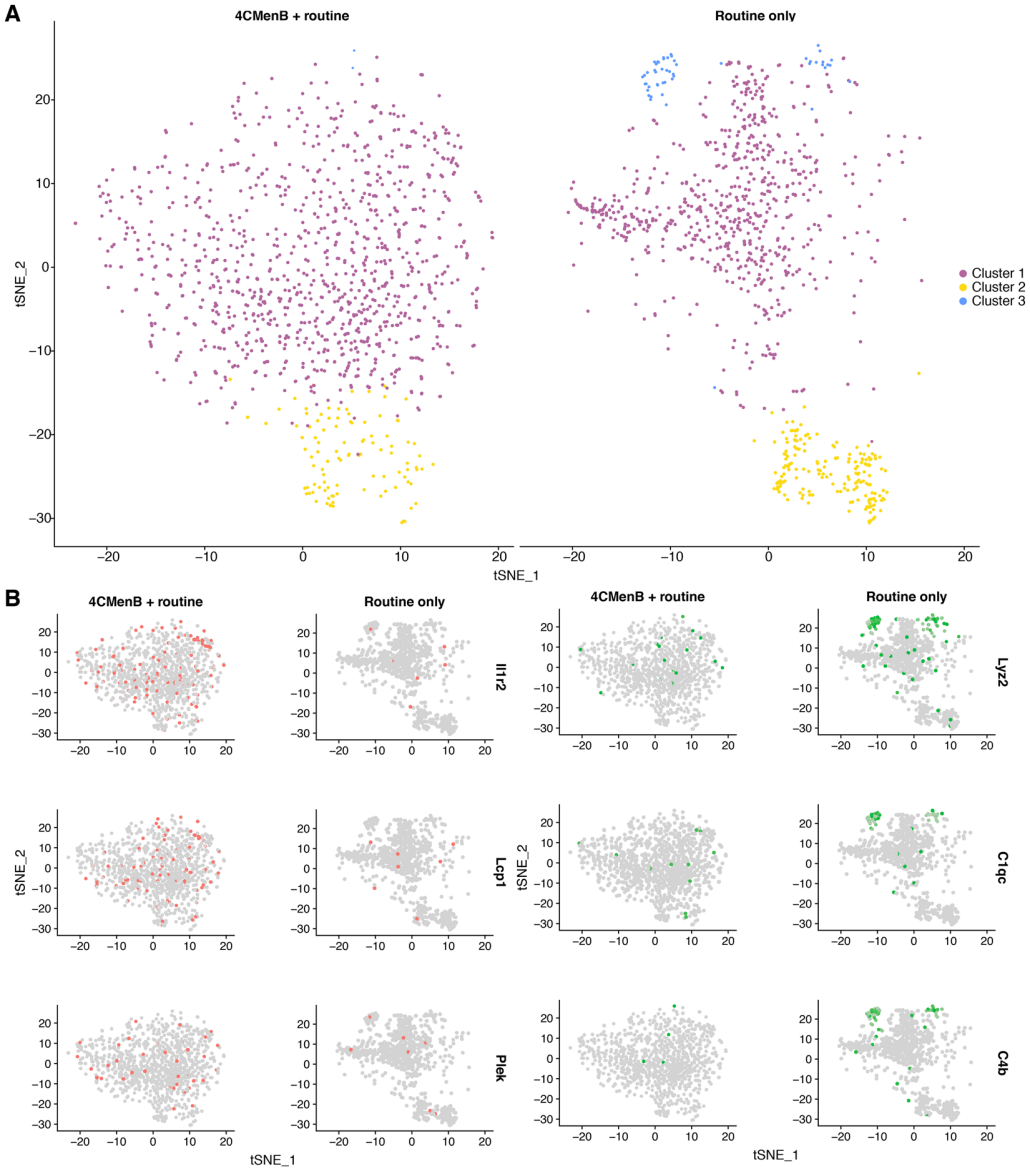
T-distributed stochastic neighbour embedding plots generated from canonical correlation analysis of neutrophils isolated from mouse whole blood, 24 hours after immunisation. (**A**) Clustering of canonical correlation analysis (CCA)-aligned neutrophils, isolated and sequenced from pooled whole blood of mice (n = 6 per group) taken 24 hours after the second dose of 4CMenB + routine and routine only immunisations. Neutrophil clusters were determined by t-distributed stochastic neighbour embedding (tSNE) dimensionality reduction during integrated analysis of CCA-aligned cells from both conditions. (**B**) Feature plot maps of top genes identified by differential expression analysis for each condition, with cells highlighted based on scaled expression values greater than one log_2_ fold change above the median across all cells. The cells highlighted in red correspond to those expressing the top genes, *Il1r2*, *Lcp1*, *and Plek*, identified as positively differentially expressed in the 4CMenB + routine group relative to the routine only group and those highlighted in green correspond to those expressing the top genes, *Lyz2*, *C1qc*, *C4b*, identified as positively differentially expressed in the routine only group relative to the 4CMenB + routine group.

Given the number of neutrophil-specific genes found among the top significantly DEGs at 24 hours in the 4CMenB and OMV groups, and the similar enrichment of the neutrophil fraction of whole blood in the 4CMenB + routine group in the infant study, we sought to determine whether concomitant immunisation led to differential transcriptional activity of neutrophils in concomitant- or routine-immunised mice. To this end, single cell RNA-seq (scRNA-seq) was performed on neutrophils isolated from mice immunised with 4CMenB + routine or routine vaccines only, 24 hours after the second dose (Supplementary Figs 5 and 6). Cells from each condition underwent canonical correlation analysis (CCA) to identify variation between conditions and facilitate an integrated analysis of all cells. A t-distributed stochastic neighbour embedding (tSNE) clustering analysis was performed to highlight differences and similarities between cells and showed a high degree of overlap between clusters from each condition (Fig. 8A). Comparative analysis of DEGs between conditions identified the innate immune receptor genes *Il1r2* to be distinctly upregulated in the 4CMenB + routine group, while the lysozyme-encoding *Lyz2* and complement genes *C1qc* and *C4b* underwent greater upregulation in the routine only group (Fig. 8B). Also among the top DEGs in the 4CMenB + routine group were *Plek* and *Lcp1*, the two genes responsible for the greatest contribution to the clustering of OMV groups in the RNA-seq PCA.

### Pathway enrichment analysis of significantly differentially expressed genes in whole blood

Taking lists of the top significantly DEGs identified for each group, pathway over-representation analyses (ORA) were conducted using curated public databases of genes characterised for specific ontologies. A common set of significantly enriched innate immune response pathways was identified for the OMV-containing vaccine groups and the related *E*. *coli* LPS group (Supplementary Fig. 7) defined by TLR4 activation, with the notable exception of the lpxL1 nOMV group, and (NLRP3) inflammasome activation. Cytokine/chemokine-specific ORA indicated that the IL-6 signalling pathway was significantly enriched for all groups, with the exception of the viral control (Supplementary Fig. 8). Additionally, several type 2 helper T cell (T_H_2) cytokine signalling pathways, including IL-4, IL-5, IL-6, and IL-13 were significantly enriched among the OMV groups. This may be partly explained by the fact that alum stimulates a T_H_2 cytokine response^23^. Significant enrichment of IL-1 was associated with the 4CMenB and OMV groups. An ontology found to be significantly downregulated in the NHBA and NadA protein groups, but not the fHbp group, was the cytochrome P450 (CYP) pathway. A clustering analysis of the genes associated with this family highlighted that these genes were highly and consistently downregulated only in the NHBA and NadA groups (Supplementary Fig. 9). The CYP gene families play an important role in the metabolism of arachidonic acid, the precursor of prostaglandin E2 (PGE_2_), encoding enzymes that convert it to eicosanoid metabolites^24^. Their expression in hepatocytes is known to be regulated by IL-6 during the inflammatory response, leading to their downregulation^25^.

### IL-6 levels are significantly elevated after immunisation with 4CMenB and its components

**Figure 9:**
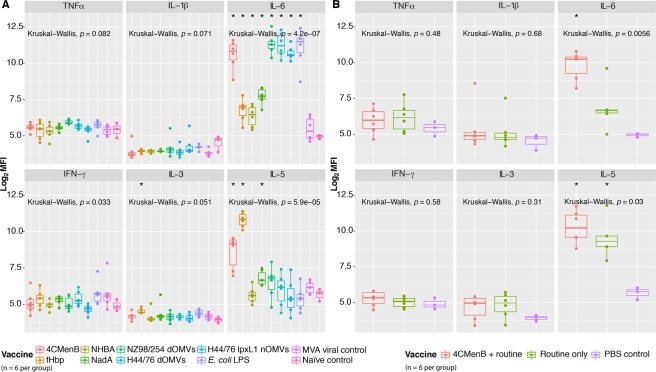
Detection of cytokines in mouse sera. Log_2_ median fluorescence intensity (MFI) values for each of cytokine detected by a six-plex cytokine bead assay 24 hours after immunisation with (**A**) 4CMenB, one of its constituent components, or one of several comparator antigens, or (**B**) 4CMenB + routine, routine only, or PBS control immunisations. Kruskal-Wallis significance values are presented in the top left corner of each panel. Significance of group medians relative to naïve or PBS controls were calculated using Mann-Whitney rank-sum tests. *< 0.05.

To determine the validity of the cytokine pathway ORA, a multiplex cytokine bead assay was performed on sera separated from whole blood 24 hours after the second dose of each vaccine/antigen (Fig. 9A). IL-6 was significantly elevated for all groups, with the exception of the viral control, as suggested by the pathway ORA. The highest IL-6 levels were observed in the 4CMenB- and OMV- immunised groups. Significant quantities of TNFα were detected in the NZ98/254 OMV immunised group and a slight increase occurred in the *E*. *coli* LPS- immunised group, also suggested by the pathway ORA. While the changes in IL-1β levels did not reach significance, there was an observable increase in the *E*. *coli* LPS- immunised group and several outliers in the 4CMenB- and OMV- immunised groups. A significant increase in IL-3 was detected in the fHbp- immunised group, and a significant increase in IL-5 was detected for the 4CMenB-, fHbp-, and NadA- immunised groups, with the highest levels seen in the fHbp- immunised group. These cytokines, along with GM-CSF, make up a cytokine family that has roles in the differentiation, recruitment, and growth of immune cells, and are characteristic of a T_H_2 response^26^. The fact that alum is included in all vaccines/antigens, except MVA, yet significant levels of this cytokine were not detected for across all groups indicates that this is an antigen-specific rather than adjuvant-induced response.

The same multiplex assay was performed on sera from mice after receiving 4CMenB + routine, routine only, or PBS control immunisations (Fig. 9B). IL-6 was the only significantly elevated cytokine in the test groups, with greater quantities detected in the concomitant group than the routine group. There was also an increase in the levels of IL-5 in both of these groups, relative to the PBS controls. Several other cytokines were detected but did not present changes that reached significance.

### Cytokine signal transducer and prostaglandin-synthesising enzyme genes are significantly upregulated in brain endothelia cells after 4CMenB and OMV immunisation

**Figure 10:**
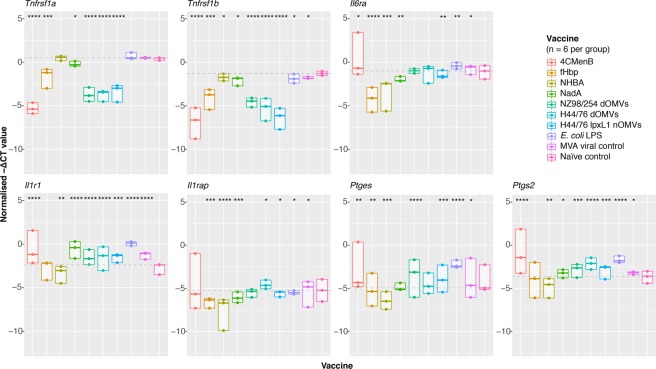
Expression of reactogenic cytokine receptor and prostaglandin enzyme genes on brain endothelial cells. Normalised (Δ) threshold cycle (C_T_) values were calculated by subtracted the mean C_T_ value for the reference gene (*Pgk1*) from the target gene C_T_ values, averaged across triplicates. These values are displayed are plotted as negative ΔC_T_ to demonstrate their logical relation to the naïve control group. The horizontal line corresponds to the median of the naïve control group for each target gene, values above this line are considered upregulated and those below are considered downregulated, relative to the naïve control. Significance values were calculated between the test and naïve control groups using a Mann-Whitney test. *< 0.05, **< 0.01, ***< 0.001, ****< 0.0001.

Among the top DEGs were several encoding a variety of innate immune cytokine receptors. A hierarchical clustering analysis of these receptors revealed their expression to be associated with 4CMenB and OMV immunisation (Supplementary Fig. 10). Several of these receptor genes, such as *Il1rap*, *Il1r2*, *Tnfsfr1a*, *Tnfsfr1b*, and *Il6ra*, encode the cognate receptors for some of the reactogenic cytokines detected to varying degrees in sera. This prompted us to explore their role in the final mediation of fever at the blood brain barrier, initiated by the binding of these cytokines to their cognate receptor on brain endothelial cells (BECs) and the subsequent induction of prostaglandin synthesis. BECs were isolated from the brains of mice immunised with 4CMenB or one of our vaccines/antigens of interest (Supplementary Fig. 11). Real-time quantitative PCR (RT-qPCR) was then performed on RNA extracted from these cells. Figure 10 shows the negative normalised (reference gene subtracted, Δ) threshold cycle (C_T_) values for each gene tested. Both of the TNFα receptor genes, *Tnfrsf1a* and *Tnfrsf1b*, were very significantly downregulated in the 4CMenB-, fHbp-, and three OMV-immunised groups. The IL-6 receptor gene, *Il6ra*, was significantly upregulated in the 4CMenB-, *E*. *coli* LPS-, and MVA- immunised groups, but was very significantly downregulated in the three protein-immunised groups. The IL-1 receptor (IL1R1) was highly significantly upregulated in all groups except the fHbp- and NHBA- immunised groups, in which it was significantly downregulated. IL1RAP was only significantly upregulated in the H44/76 dOMV- and MVA-immunised groups and was very significantly downregulated in the three protein-immunised groups. Two key PGE_2_-synthesising enzymes, microsomal prostaglandin E synthase-1 (mPGES-1) and cyclooxygenase-2 (COX-2) were highly significantly upregulated in the 4CMenB-, OMV-, and *E*. *coli* LPS-immunised groups.

## Discussion

Here we present a comprehensive analysis of the temperature, cytokine, and gene expression changes observed in mice in response to 4CMenB, administered concomitantly and on its own, and in response to its constituent antigens or several comparator antigens. The temperature data demonstrate that the human observation of exacerbated fever resulting from concomitant administration of 4CMenB with routinely-administered vaccines can be recapitulated in a mouse immunisation model. The comparison of the individual 4CMenB components, and the inclusion of OMV and *E*. *coli* LPS comparator groups provide further evidence that the endotoxin-containing OMV component of the vaccine is the causative agent of febrile responses. 4CMenB elicits a substantial perturbation in the mouse blood transcriptome 24 hours after the second dose of vaccine. Furthermore, similar changes in the expression of genes involved in immune regulation occurred in mice receiving OMVs or *E*. *coli* LPS indicate that endotoxin is the likely driver of inflammation-induced responses, with noticeable enrichment of neutrophil-related genes, subsequently characterised through single cell analysis. Finally, the systemic responses to 4CMenB and OMVs manifest in gene expression changes in BECs indicating the mechanism by which 4CMenB generates the febrile response. Taken together, these data attribute 4CMenB reactogenicity to the outer membrane composition of its constituent OMVs, providing evidence at each step of fever generation and supported by longitudinal temperature measurements.

The increased rate of fever associated with concomitant 4CMenB immunisation compared with routine vaccines or 4CMenB administered on its own has been reported in several studies^8,27^. A significant rise in surface temperature, beginning at approximately three hours after the second dose of immunisation and rising by close to 2 °C by 24 hours, occurs in mice receiving 4CMenB concomitant with routine immunisations. No significant rise in temperature was associated with routine immunisations only, in contrast with an increase reported in some infants^27^. This may be a result of the reduced dose used for mouse immunisations. The rise in temperature in mice receiving 4CMenB could be attributed to the endotoxin present in the OMV component of the vaccine. A similar rise in temperature was observed in mice immunised with other OMV-containing vaccines, though to a lesser degree in H44/76 lpxL1 nOMV-immunised mice. In its hexa-acylated lipid A form, endotoxin is a potent TLR4 ligand. Inactivation of the *lpxL1* gene results in the biosynthesis of an attenuated penta-acylated endotoxin that is known to substantially reduce pyrogenicity^28,29^, though this form of endotoxin still activates TLR4 in mice^30^. The rise in temperature associated with the dOMV vaccines was on par with that of the *E*. *coli* LPS positive control group, while the protein in alum and MVA viral control groups underwent only a mild rise in temperature relative to baseline. The attenuated endotoxin contained in the lpxL1 nOMVs does not induce a significant rise in temperature. These data suggest that the febrile response to 4CMenB and OMV vaccines is indeed driven by the hexa-acylated lipid A motif found in the endotoxin of wild-type OMV-derived preparations.

Consistent with studies comparing the adult immune response to similar stimuli with those of mice at the same time point and using the Spearman correlation of LFCs between expressed orthologs, a strong association was observed between the mouse and human infant whole blood gene expression changes induced by 4CMenB^31–33^. While the comparison does not account for the additional vaccines administered to the infant 4CMenB group, it demonstrates that mice undergo a similar transcriptional response to immunisation with a vaccine containing bacterial antigens, providing further rationale for our use of a mouse immunisation model. A gene signature defined by innate signalling pathway and neutrophil-specific genes distinguished the 4CMenB and OMV groups from the recombinant protein and viral control groups, suggesting that these components induce two very different types of responses at the transcriptional level. The presence of genes encoding receptors recognising bacterial PAMPs, *Tlr2*, *Pglyrp1*, *Tlr4*, *Tlr5*, *Tlr6*, *Tlr13*, and *Trem1*, among the most significantly upregulated genes in response to the 4CMenB and OMV vaccines indicate that these vaccines/antigens stimulate activation of immune cells in a manner similar to bacteria. Surprisingly, *E*. *coli* LPS did not induce TLR4 expression to the same degree as endotoxin-containing vaccines and appeared to activate TLR2 and other PRRs. One of the purposes of the adsorption of OMV-containing vaccines on alum is to minimise the amount of free/non-membrane-bound endotoxin, so it follows that adsorbing *E*. *coli* LPS directly on alum may dampen TLR4 stimulation^11^. The activation of TLR2 and other PRRs by *E*. *coli* LPS is likely due to the fact that the commercial preparation used is known to contain contaminant PAMPs from the bacterial cell wall and outer membrane, including peptidoglycan^34^. Interestingly, TLR2 and PGLYRP-1 are both activated by peptidoglycan, and in its peptidoglycan-bound form, PGLYRP-1 also activates TREM-1^35,36^. TREM-1 has been identified as an amplifier of endotoxin-induced septic shock, suggesting a potential mechanism for the synergistic activation of pyrogenic pathways through endotoxin and peptidoglycan innate signalling pathways^37^. The signalling events initiated by the binding of these bacterial PAMPs were further reflected by the expression of the gene encoding the NLRP3 inflammasome, *Nlrp3*, and the expression of *Il1b* which encodes the highly proinflammatory cytokine produced by inflammasome activation^38^. While the NLRP3 inflammasome is also known to be triggered by alum, the same degree of upregulation was not observed for the protein-in-alum groups, indicating that it is mainly triggered by the OMVs^39^.

The high representation of neutrophil-specific genes among the top DEGs in the whole blood transcriptomics analysis, despite their low relative abundance in mice^40^, encouraged further exploration of vaccine-specific responses in these cells. Neutrophils are underrepresented in scRNA-seq data as there are technical difficulties associated with their isolation, stability, and preservation that existing pipelines are not yet fully suited to handling^41^. The neutrophils sequenced in the present study yield information about the neutrophil-specific response to immunisation and indicate transcriptional divergence at the single cell level in mice who received concomitant administration of 4CMenB when compared with the routine group. It is interesting to note that the two genes responsible for the clustering of OMV groups in the whole blood transcriptomic PCA, *Lcp1* and *Plek*, were among the most expressed genes in the concomitant group neutrophils, indicating that the OMV component of 4CMenB may be responsible for this transcriptional activity.

Finally, the stimulation of cognate receptors on BECs at the blood-brain-barrier by the circulating proinflammatory cytokines IL-6 and IL-1β is known to induce prostaglandin synthesis and is the accepted model of inflammation-induced fever^42^. TNFα also contributes to inflammation-induced fever by stimulating the production of other cytokines^43^. The expression of the receptors for these pyrogenic cytokines and the key PGE_2_-synthesising enzymes on BECs was quantified. Significant upregulation of mPGES-1 and COX-2 on BECs from 4CMenB- or OMV/*E*. *coli* LPS-immunised mice indicates that these antigens elicit the strongest indicators of fever at the neurological level. These results are consistent with a study examining the BEC transcriptional response to LPS-induced fever where the expression of the genes *Il1r1*, *Il6ra*, *Ptges*, and *Ptgs2* were significantly upregulated^44^. Contrary to their findings however, the TNFα receptor genes *Tnfrsf1a* and *Tnfrsf1b* were found to be significantly downregulated in response to 4CMenB or OMV-immunisation, suggesting that this cytokine may be produced rapidly and transiently following immunisation, thereby downregulating expression of its receptor through a homeostatic response, or may play an indirect role in the mediation of the febrile responses to these vaccines by stimulating the production of IL-1β and IL-6 from other cells.

## Conclusions

These data show that a mouse model is suitable for study of reactogenicity of vaccines with comparable increases in fever with concomitant 4CMenB immunisation, as compared with routine immunisation, and corresponding transcriptomic changes. Furthermore, the rise in temperature associated with this vaccine can be attributed to the endotoxin-containing OMV component of the vaccine and not the recombinant proteins. High-throughput sequencing allowed for the identification of a distinct bacterial gene expression signature that distinguished the transcriptional response to OMVs from that of the other 4CMenB constituents. This response is primarily elicited by the endotoxin found in these vaccines, but could also be exacerbated by other bacterial outer membrane antigens, and results in the upregulation of neutrophil-specific inflammatory markers. The systemic responses induced by 4CMenB and wild-type OMVs are transduced at the blood brain barrier, manifesting in the upregulation of PGE_2_ synthesis genes that mediate the final stages of fever induction. These data demonstrate that mice immunised with OMV-containing vaccines exhibit the immunological hallmarks of fever and the presence of hexa-acylated endotoxin determines their pyrogenicity.

## Methods

### Ethics statement

All procedures were performed in accordance with the terms of the UK Home Office Animals Act Project License. The University of Oxford Animal Care and Ethical Review Committee approved procedures.

### Vaccines and antigens

4CMenB (Bexsero®, GlaxoSmithKline), DTaP-IPV-Hib (Pediacel®, Sanofi Pasteur), PCV13 (Prevnar 13®, Wyeth Lederle Vaccines S.A.), and PBS (Sigma-Aldrich) were used for routine versus concomitant experiments. 50 μg of each of the three 4CMenB recombinant proteins – fHbp, NadA, and NHBA (Bexsero®, GlaxoSmithKline) – were diluted in PBS and combined with an equal volume of alum (Sigma-Aldrich). 25 μg of NZ98/254 dOMVs (GlaxoSmithKline), H44/76 dOMVs (Oxford Vaccine Group), and lpxL1 nOMVs (Oxford Vaccine Group) were prepared in the same manner. *E*. *coli* LPS (from *E*. *coli* O111:B4, Invivogen) was diluted in PBS to a concentration of 5 μg/mL and diluted 1:2 in alum. MVA (Oxford Vaccine Group) was diluted in PBS to a concentration of 5 × 10^8^ infectious units per mL.

### Mouse immunisations

Immunisations were performed under general anaesthesia, using six–eight-week old female C57BL/6 (Harlan) mice (6 per group). A maximum total volume of 100 μL of vaccine/antigen was administered to each animal intramuscularly (IM) in accordance with the Project License, hence vaccines with a 500 μL administration volume for humans were restricted to 1/5 of the human dose in mice or 1/15 of the human dose if administered as part of a three-vaccine combination. For experiments testing a single vaccine/antigen per group, 50 μL of vaccine was injected into each leg of each mouse and for experiments testing three-vaccine combinations 33 μL of each vaccine was injected into the left, lower right, and upper right thigh of each mouse. Intraperitoneal immunisation would have allowed for a higher dose of vaccine, but IM immunisation was preferable to mimic the human infant immunisation. Vaccine/antigens were administered 21 days apart. Cardiac bleeds were performed 24 hours after the second dose of vaccine. Blood for RNA-seq experiments was transferred to RNAprotect® Animal Blood Tubes (QIAGEN) containing RNA-stabilising reagent and incubated at room temperature for two hours to lyse blood cells. For neutrophil isolation, blood was transferred to cryovials containing of heparin sodium (Fannin) anti-coagulant at a ratio of 15 μL per mL of blood.

### Mouse temperature measurement

Mouse surface temperatures were measured in triplicate by infared thermometry using a No Touch Thermometer NTF3000 (Braun), as outlined by Mei *et al*.^45^, and averaged to obtain time point temperature readings for each mouse.

### Brain endothelial cell isolation

Mouse brains were harvested, enzymatically digested, and stained for FACS isolation of BECs according to the protocol developed by Crouch and Doetsch^46^. 4′,6-diamidino-2-phenylindole (DAPI, Life Technologies), anti-CD31-APC (clone: MEC 13.3) and anti-CD45-PE (clone: 30-F11) antibodies (BD Biosciences) were used to isolate CD31^+^CD45^−^ live cells (Supplementary Fig. 11). Cells were sorted directly into 2 mL microcentrifuge tubes, containing 350 μL of RLT buffer (QIAGEN) containing β-mercaptoethanol at a concentration of 1:50, and stored on ice. RNA was extracted from sorted cells using a RNeasy Plus Micro Kit (QIAGEN) and stored at −20 °C.

### Real time quantitative PCR

BEC RNA was reverse transcribed to cDNA using a High-Capacity cDNA Reverse Transcription Kit (ThermoFisher) and pre-amplified using pooled TaqMan™ primers (Supplementary Table 1) at a concentration of 0.4X and a TaqMan™ PreAmp Master Mix Kit (ThermoFisher). Thermocycling parameters can be found in Supplementary Table 2A. Pre-amplified cDNA samples were diluted 1:20 in 1X TE buffer (Invitrogen) and combined in the appropriate ratio with TaqMan™ Gene Expression Master Mix (ThermoFisher), nuclease-free water, and the appropriate primer on a MicroAmp Fast Optical 96-Well Reaction Plate (0.1, ThermoFisher). Samples were assayed in triplicate for each primer and RT-qPCR was performed using a StepOnePlus™ Real-Time PCR System (ThermoFisher). Thermocycling parameters are can be found in Supplementary Table 2B. The data obtained were normalised by subtracting the ΔC_T_, value averaged across triplicates, for the reference gene (*Pgk1*) from that of the target gene for test and control groups.

### RNA sequencing

RNA was extracted from mouse whole blood samples using a Mouse RiboPure™-Blood RNA Isolation Kit (Life Technologies) and depleted of globin mRNA using a GLOBINclear™ mouse/rat Kit (Life Technologies). RNA integrity was measured using a 2100 Bioanalyzer Instrument (Agilent Technologies). Polyadenylated transcripts were oligo (dT) bead-selected, reverse transcribed, amplified and labelled with an Illumina TotalPrep™-96 RNA Amplification Kit (Life Technologies). Sequencing was conducted at the Wellcome Centre for Human Genetics (University of Oxford) where sample fragments were 75 bp size-selected and multiplexed prior to paired-end sequencing with an Illumina HiSeq4000 system (Illumina).

### RNA-seq analysis

Quality control (QC) of raw reads was performed using FastQC software (v0.11.8)^47^. The genome index was built using the *Mus musculus* GRCm38 reference genome and annotation (release 95, https://www.ensembl.org/Mus_musculus/Info/Index) using STAR v2.7^48^. Read counts were estimated at the gene level concurrently with read alignment using STAR’s ‘-quantMode GeneCounts’ function. The resultant count matrices were analysed using R (v3.8) and assessed for differential gene expression (DGE) using a combination of edgeR (v3.8) and limma/voom (v3.8)^49–53^. Lowly-expressed genes were filtered using a threshold of counts per million values >3 in *N* libraires, where *N* is the smallest group sample size, and normalised using the trimmed mean of M-values method^54^. Linear models were fitted to the voom-transformed data and genes were ranked for DE by empirical Bayes testing of the fitted model^55^. Significantly differentially expressed genes were defined as those with an FDR-adjusted *p*-value of less than 0.01 and an absolute LFC value >1.2. gene ontology and pathway ORA was conducted using the InnateDB online database and selecting the Reactome and INOH pathway annotations^56^. To compare the similarity of gene expression changes induced by a 4CMenB-containing vaccine regimen between the mouse and human infant datasets, the Spearman rank correlation coefficient of the log_2_ fold changes across all one-to-one orthologs expressed in at least one species was calculated.

### Neutrophil isolation

Serum was separated from heparin-stabilised blood tubes by microcentrifugation at maximum speed for ten minutes. Sera were stored at −20 °C for cytokine analysis. Pellets were resuspended in 1X BD FACS™ Lysing Solution (BD Biosciences), transferred to a 15 mL tube (Grenier Bio-One), topped up to 10 mL with lysis solution, and left to incubate for 15 mins at room temperature. Tubes were then centrifuged at 500 × *g* for ten minutes. Supernatants were discarded and pellets were washed twice by resuspending in 1 mL of autoMACS® rinsing solution (Miltenyi Biotec), centrifuging at 300 × *g* for 10 minutes, and discarding the supernatant. After the second wash was complete, the pellet was resuspended in 100 μL rinsing buffer and the appropriate concentration of antibodies were added: 1:200 PE Rat Anti-Mouse Ly6C (clone: AL-21), 1:10 BV711 Rat Anti-Mouse Ly6G (clone: 1A8), 1:10 V450 Rat Anti-Mouse CD11b (clone: M1/70), 1:50 BD Horizon™ Fixable Viability Stain 780 (all from BD Biosciences). Samples were incubated in the dark at 4 °C for 30 mins, washed twice with rinsing buffer, transferred to 5 mL Falcon™ Round-Bottom Polystyrene Tubes (Corning) and then sorted using a BD FACSAria™ (BD Biosciences) cell sorter. Samples from each group (n = 6 per group) were pooled together to obtain a single sample representing a group average for sequencing. Neutrophils were identified as Ly6C^−^CD11b^+^Ly6G^+^ live cells (Supplementary Fig. 6). These cells were sorted directly into 2 mL microcentrifuge tubes (Eppendorf) containing 2% fetal bovine serum (Sigma-Aldrich) in 1X PBS and transferred to ice. A Muse® Count &Viability Assay Kit (Millipore) was used to measure the final cell count and viability of each sample on a Muse® Cell Analyzer instrument (Millipore).

### Single cell profiling

Droplet-based single-cell partitioning and scRNA-seq libraries were generated using the Chromium Single-Cell 3′ Reagent v3 Kits (10X Genomics) as per the manufacturer’s protocol. Briefly, a maximum volume (46.4 μL) of single-cell suspensions at densities of 100–350 cells/μL were mixed with RT master mix and immediately loaded together with Single-Cell 3′ Gel Beads and Partitioning Oil into a Single-Cell Chip B. The chip was then loaded onto a Chromium Controller (10X Genomics) for single-cell GEM (gel bead-in-emulsion) generation and barcoding. Upon portioning, the Gel Beads release unique oligos containing 10X cell barcodes, unique molecular identifiers (UMIs) and poly(dT) sequences. RNA transcripts from single cells were reverse-transcribed within droplets to generate barcoded full-length cDNA. cDNA molecules from each sample were recovered and amplified. Finally, amplified cDNA was fragmented, and adapter and sample indices were incorporated to make libraries compatible with Illumina sequencing. The size profiles of the amplified cDNA and sequencing libraries were assessed by an Agilent 2200 TapeStation using High Sensitivity D5000 and D1000 ScreenTapes, respectively (Agilent Technologies). Indexed libraries were pooled in equimolar ratios and sequenced on the Illumina HiSeq 4000 system with a customized paired end (28,8,98 bp) format according to the recommendation by 10X Genomics.

### Single cell analysis

BCL files were demultiplexed and converted for fastq format using the bcl2fastq function in Cell Ranger (v3.0.2, 10X Genomics)^57^. QC of fastq files was performed using FastQC software (v0.11.8)^47^. QC’d reads were then processed using the Cell Ranger pipeline to generate feature-barcode matrices. The resultant output matrices were first analysed individually using Seurat (v3.0)^58^. Lowly-expressed genes, those expressed in fewer than three cells, were removed and cells expressing greater than 20% mitochondrial transcripts were also removed as this is an indication of poor viability. After filtering, 3109 cells and 1185 cells were left to be analysed from the 4CMenB and 4CMenB + routine samples, respectively. Global-scaling normalisation was performed using the ‘LogNormalize’ function. PCA was used for dimensionality reduction and the principal components were then used for tSNE clustering. CCA was performed separately to allow for integrated analyses to be conducted using both conditions. Subspaces were aligned using condition as the grouping factor and tSNE was applied to generate clusters using the CCA-aligned cells. Conserved and differentially expressed markers were then determined between conditions.

### Cytokine quantification

A six-plex mouse cytokine/chemokine panel consisting of IFNγ, TNFα, IL-1β, IL-3, IL-5, IL-6 (Millipore) was used to quantify pro-inflammatory cytokines in mouse sera 24 hours after the second dose of vaccine. The assay was run according to manufacturer’s instruction and MFI values were obtained using a Luminex MAGPIX® instrument with Exponent software (Invitrogen).

## Supplementary information

Dataset 1

## Data Availability

All datasets have been deposited in the NCBI GEO database, with the accession numbers: GSE131929 (EUCLIDS human infant RNA-seq data), GSE131914 (4CMenB individual components mouse RNA-seq data), and GSE132199 (concomitant versus routine mouse neutrophil scRNA-seq data).
