# Supplementary material for: Comparative transcriptomics between species attributes reactogenicity pathways induced by the capsular group B meningococcal vaccine, 4CMenB, to the membrane-bound endotoxin of its outer membrane vesicle component
Source: Sci Rep. 2019 Sep 24;9:13797. doi: 10.1038/s41598-019-50310-0 (PMC6760121; doi:10.1038/s41598-019-50310-0)
Supplement: Supplementary file 1 — Dataset 1 [file 41598_2019_50310_MOESM1_ESM.pdf]

Comparative transcriptomics between species attributes reactogenicity pathways induced by the capsular group B meningococcal vaccine, 4CMenB, to the membrane-bound endotoxin of its outer membrane vesicle component

Dylan Sheerin[1,*], Daniel O'Connor[1], Christina Dold[1], Elizabeth Clutterbuck[1], Moustafa Attar[2], Christine S Rollier[1], Manish Sadarangani[3,4], Andrew J Pollard[1]

[1] Oxford Vaccine Group, Department of Paediatrics, University of Oxford, and the NIHR Oxford Biomedical Research Centre, Centre for Clinical Vaccinology and Tropical Medicine, Churchill Hospital, Oxford, UK.

[2] Wellcome Centre for Human Genetics, University of Oxford, Oxford, UK.

[3] Vaccine Evaluation Center, BC Children's Hospital Research Institute, Vancouver, BC, Canada.

[4] Department of Pediatrics, University of British Columbia, Vancouver, BC, Canada.

* To whom correspondence should be addressed. Tel: +44(0)1865 11335; Email: dylan.sheerin@paediatrics.ox.ac.uk

Table of contents

- **4CMenB immunisation increases temperature in mice when administered on its own or concomitantly with routine immunisations**
  - SF. 1. Routine versus concomitant immunisations.
  - SF. 2. 4CMenB individual components immunisations.
- **Gene signatures induced by 4CMenB and OMVs are defined by bacterial innate response genes**
  - SF. 3. Toll like receptor genes.
  - SF. 4. Signalling molecule genes.
- **Concomitant 4CMenB immunisation stimulates proinflammatory gene expression in a cluster of activated neutrophils**
  - SF. 5. Gating strategy for neutrophil isolation.
  - SF. 6. Quality control metrics of neutrophil 10x data.
- **Pathway enrichment analysis of significantly differentially expressed genes in whole blood**
  - SF. 7. Reactome over-representation analysis.
  - SF. 8. INOH over-representation analysis.
  - SF. 9. Hierarchical clustering of cytochrome P450 genes.
  - SF. 10. Hierarchical clustering of cytokine/chemokine receptors.
- **Cytokine signal transducer and prostaglandin synthesising enzyme genes are significantly upregulated on brain tissue after 4CMenB and OMV immunisation**
  - SF. 11. Gating strategy for brain endothelial cell isolation.
  - ST. 1. Primers used for real-time quantitative PCR of brain endothelial cells.
  - ST. 2. Thermocycling parameters.

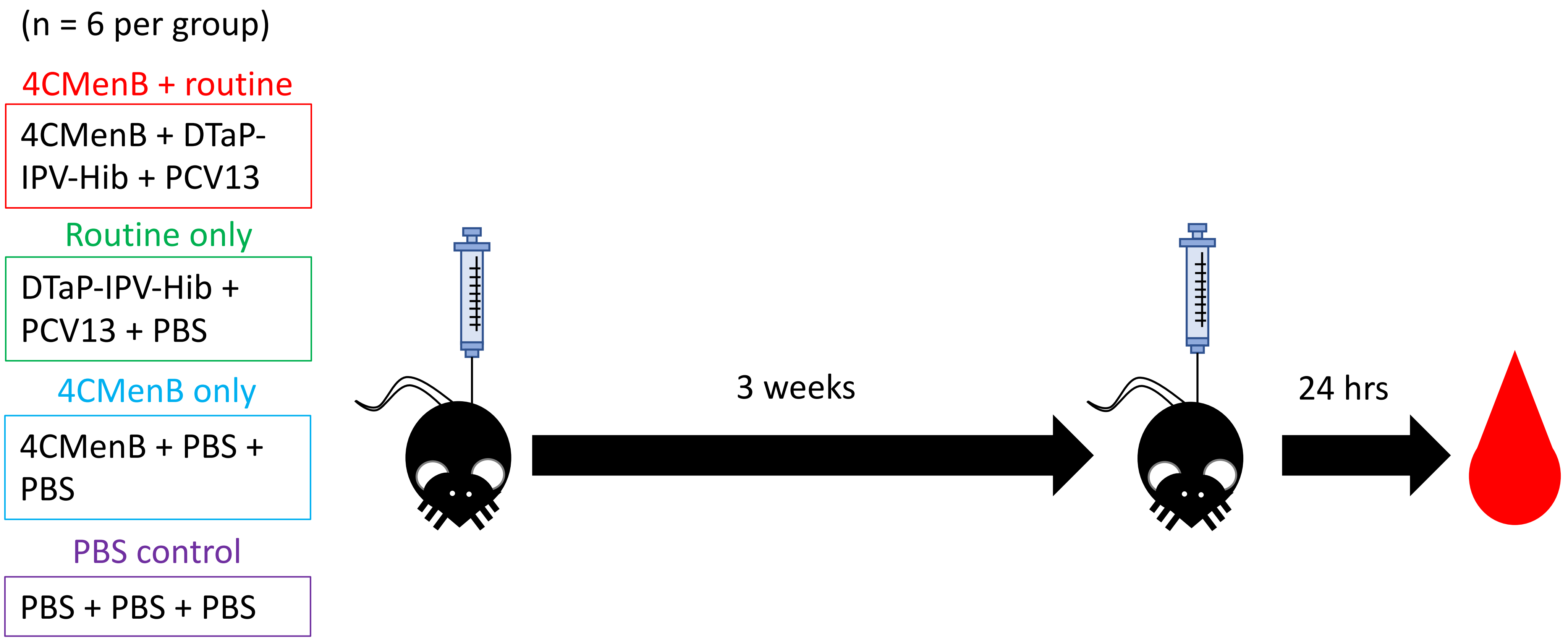

Supplementary Fig. 1. Routine versus concomitant immunisations. Schematic of mouse immunisation strategy for concomitant 4CMenB vs. routinely-administered vaccines experiment.

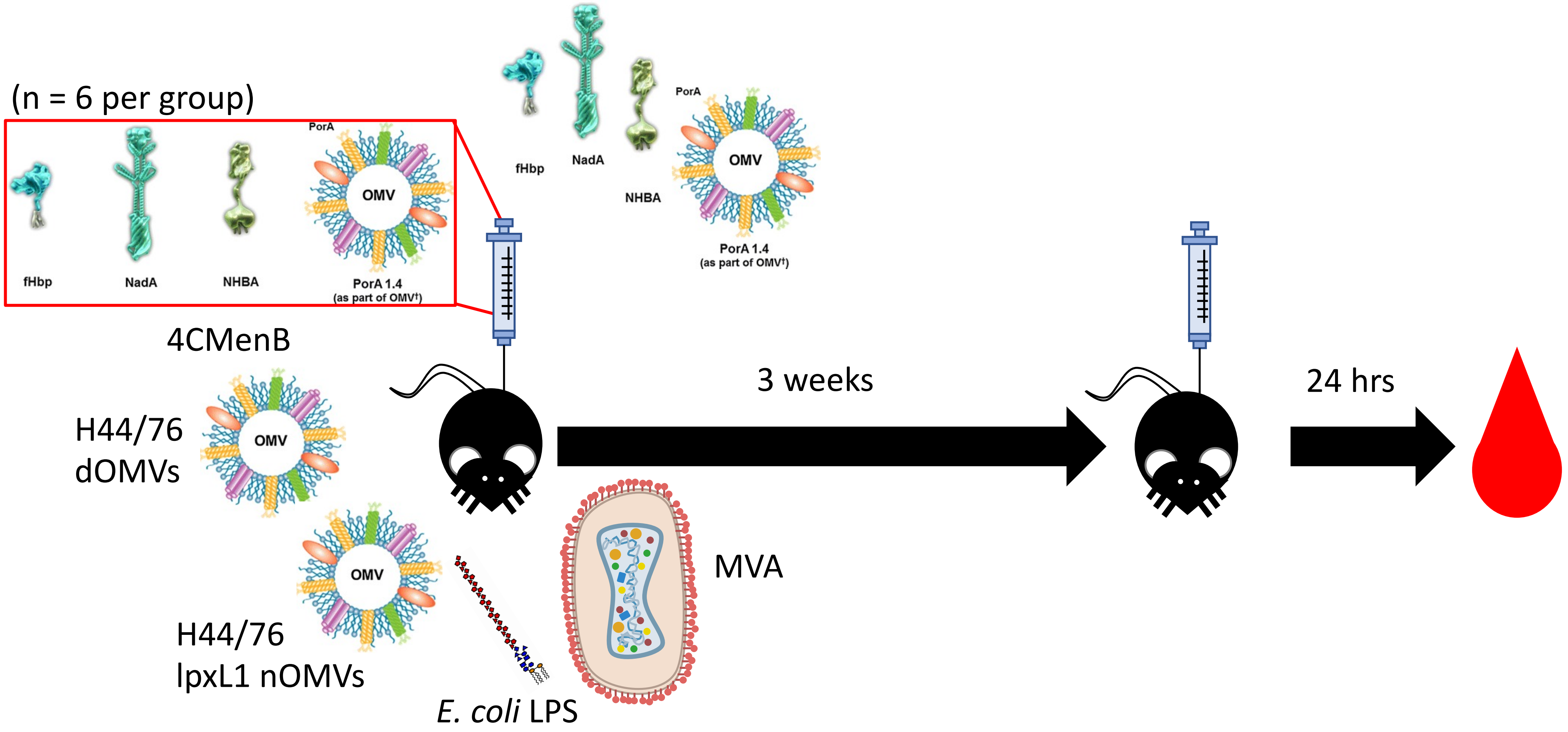

Supplementary Fig. 2. 4CMenB individual components immunisations. Schematic of mouse immunisation strategy 4CMenB, individual component, and comparator vaccine experiment. 4CMenB component figures taken from O'Ryan *et al.*, 2014.

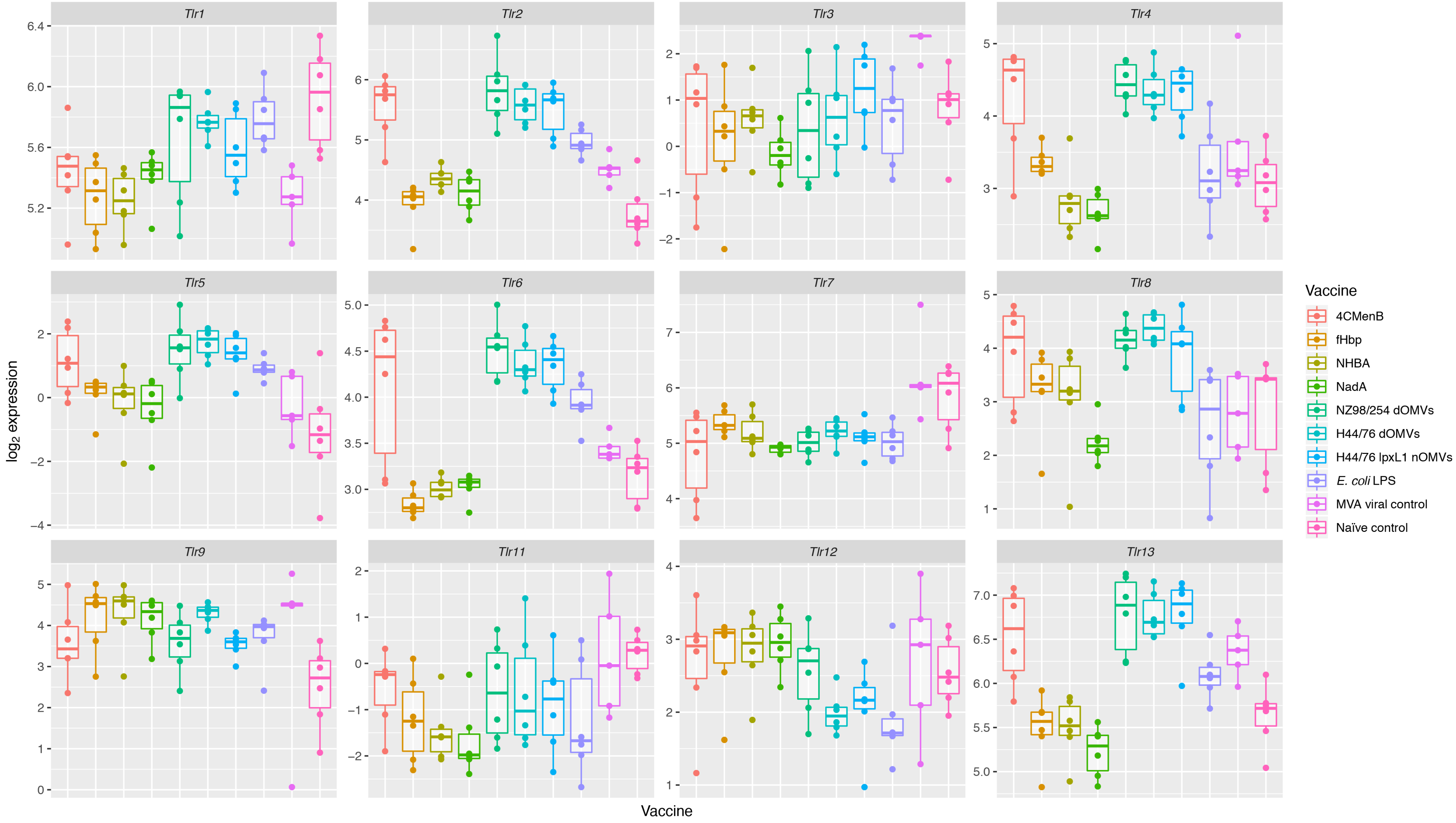

Supplementary Fig. 3. Toll like receptor genes. Log_2 normalised expression values for the mouse toll like receptor genes.

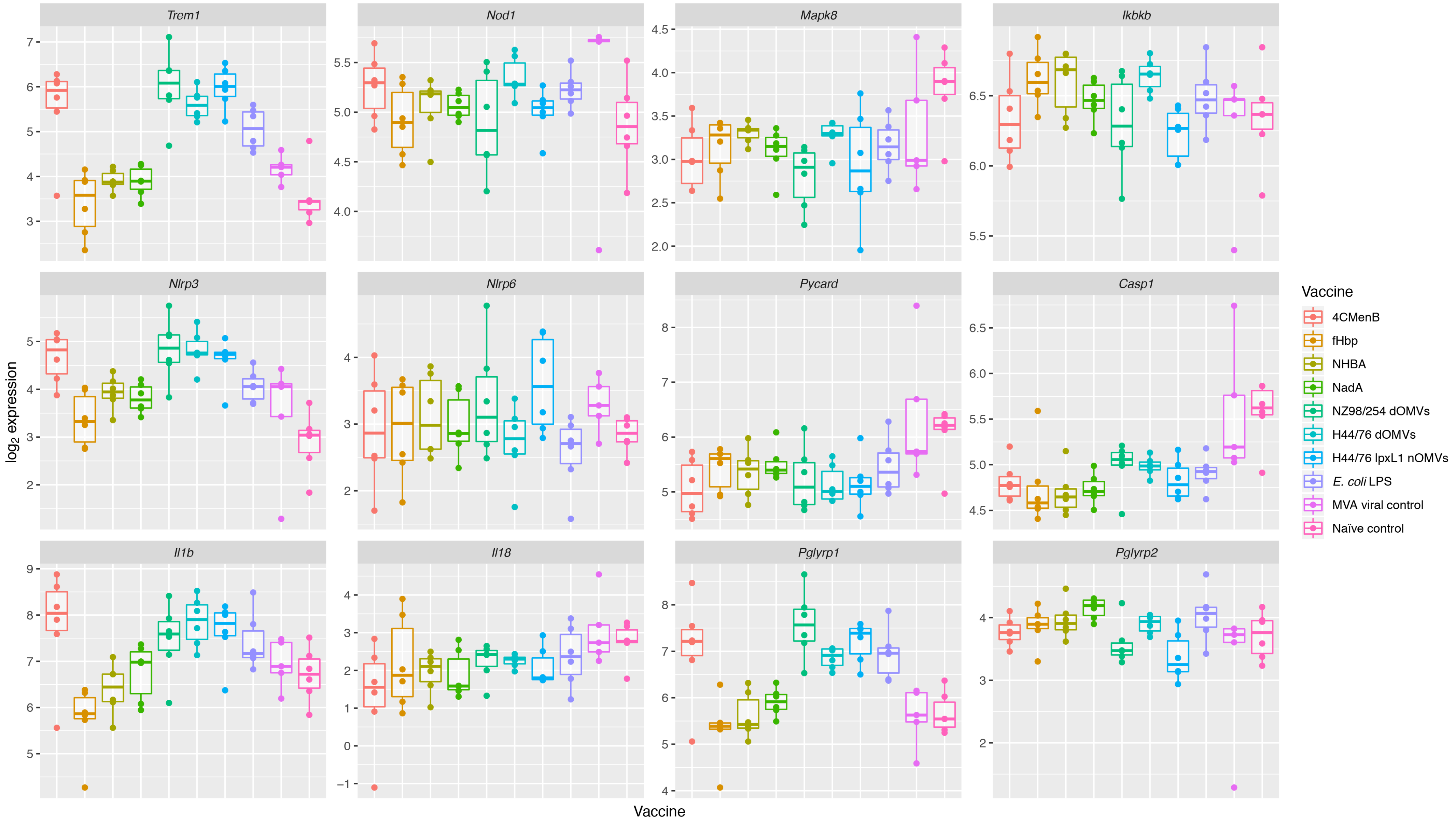

Supplementary Fig. 4. Signalling molecule genes. Log_2 normalised expression values for accessory and downstream signalling molecule genes.

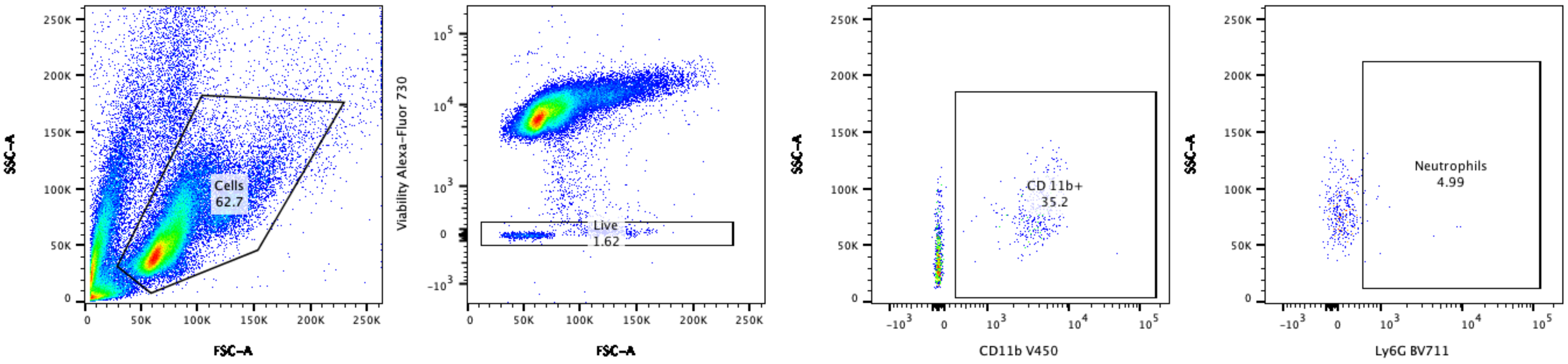

Supplementary Fig. 5. Gating strategy for neutrophil isolation. Neutrophils were identified as $Ly6C^{-}Cd11b^{+}Ly6G^{+}$ live cells in whole blood.

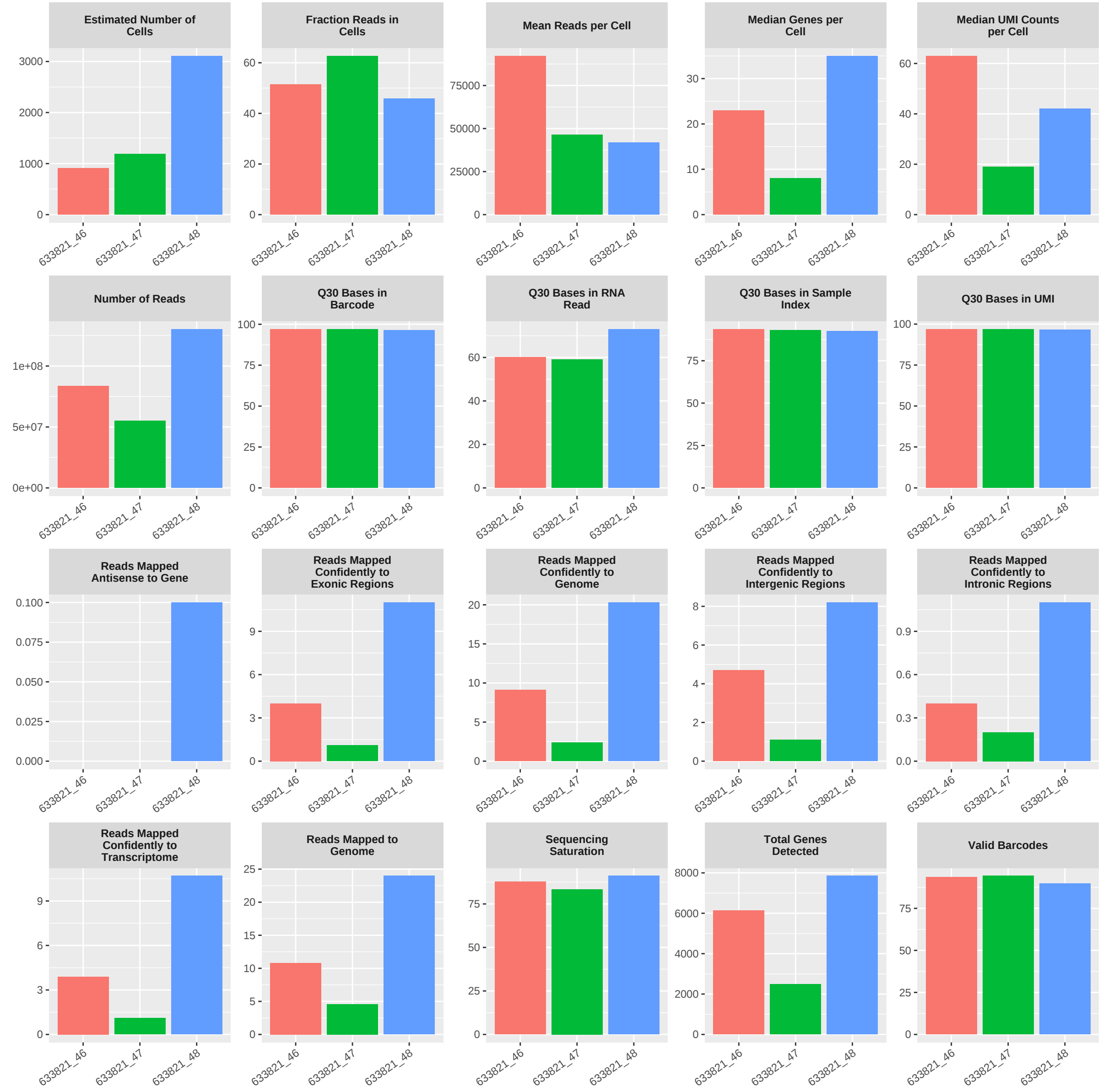

Supplementary Fig. 6. Quality control metrics of neutrophil 10x data. 633821_46 corresponds to the routine only group, 633821_47 to the PBS control group, and 633821_48 to the 4CMenB + routine group.

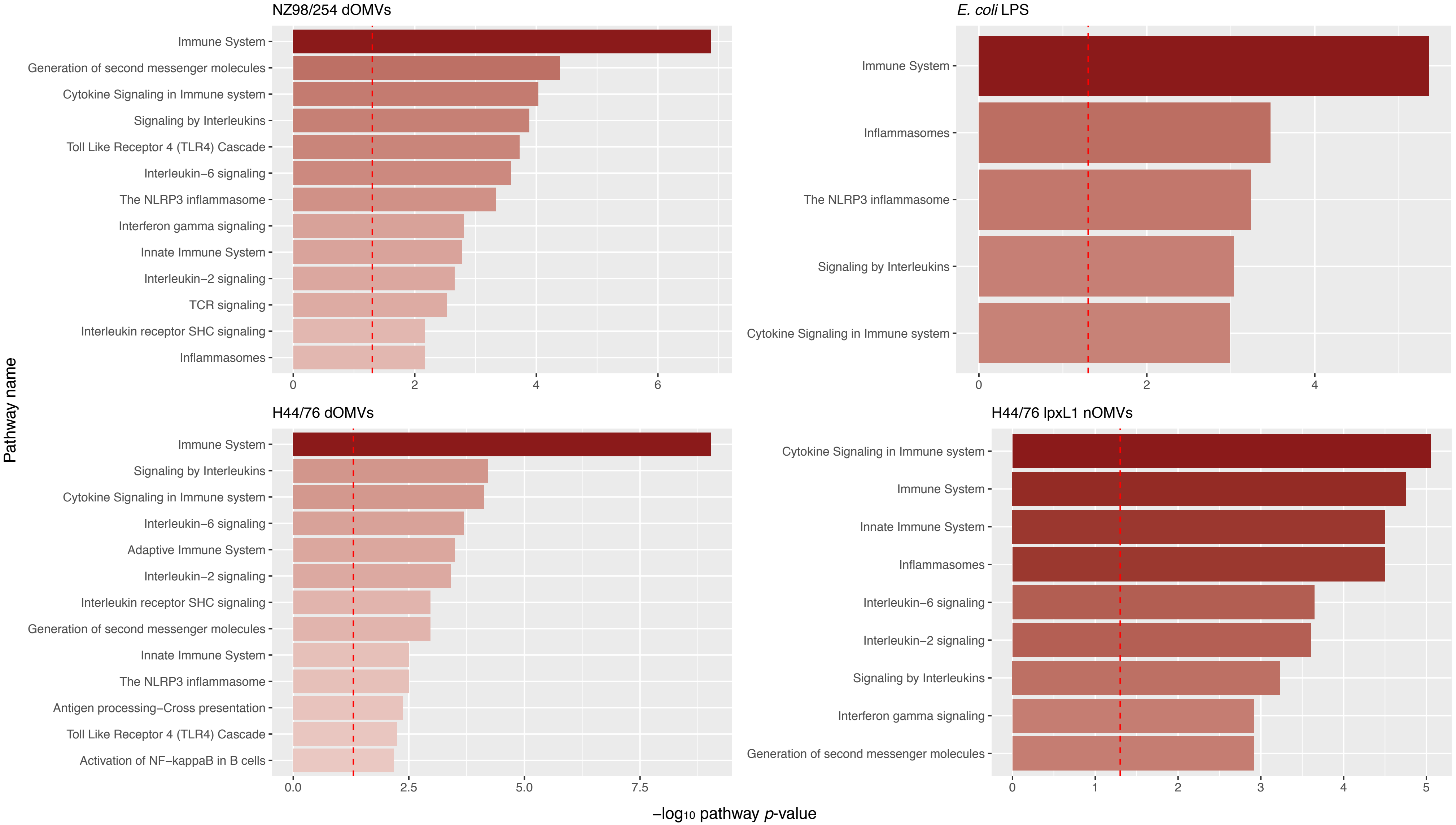

Supplementary Fig. 7. Reactome pathway over-representation analysis. Significantly enriched immunological pathway categories from the publicly available Reactome database. The NZ98/254 detergent-extracted (d)outer membrane vesicle (OMV, top left), *Escherichia coli* (*E.* coli) lipopolysaccharide (LPS, top right), H44/76 dOMV (bottom right), H44/76 lpxL1 native (n)OMVs (bottom right) groups are presented here. The dashed red line corresponds to a negative $\log_{10}$ pathway adjusted *p*-value of 0.05.

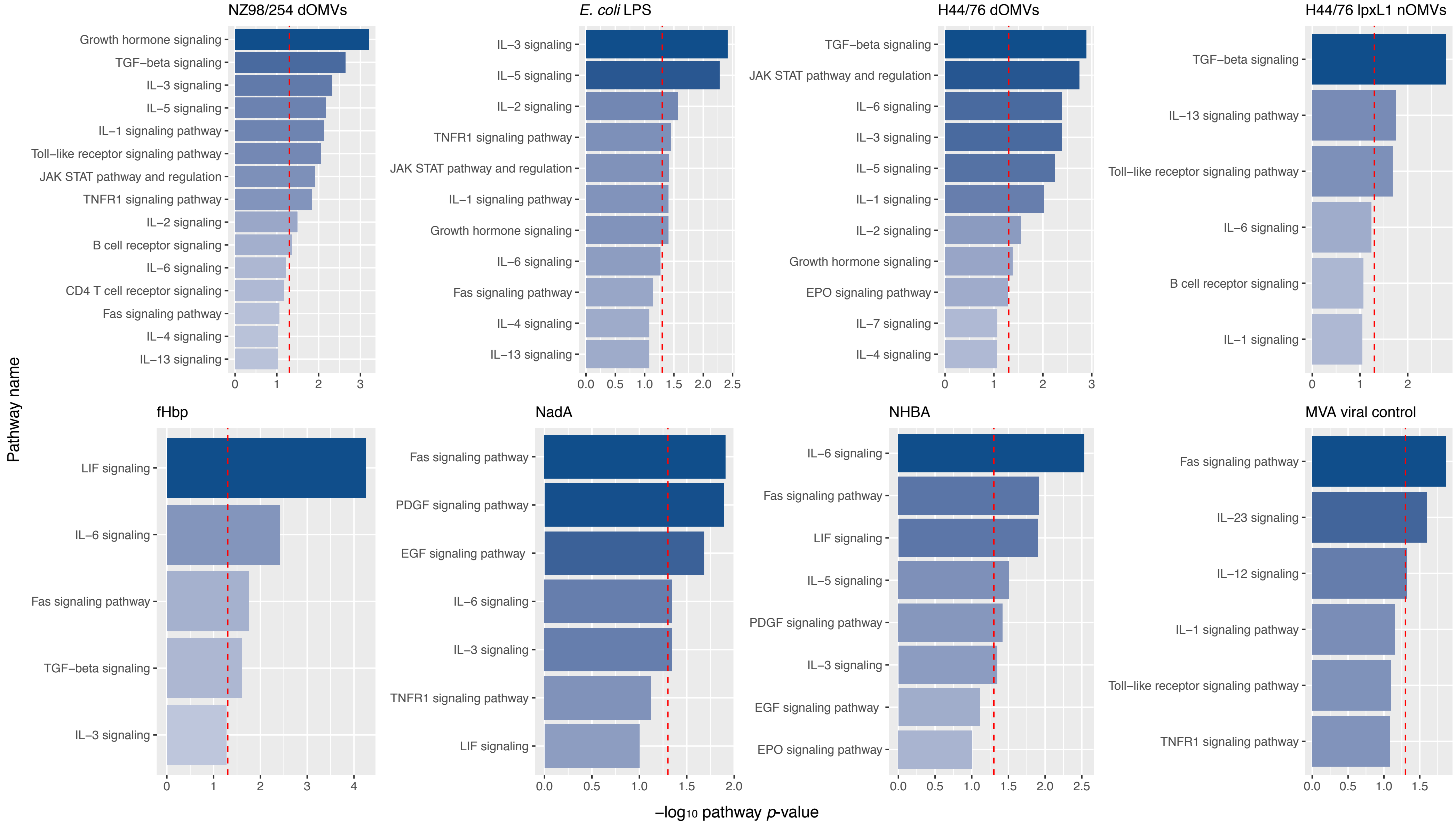

Supplementary Fig. 8. INOH pathway over-representation analysis. Significantly differentially expressed genes (FDR < 0.05) determined for each group comparison were assessed for enrichment of genes pertaining to cell signalling pathway categories from the publicly available integrating network objects with hierarchies (INOH) database. The dashed red line corresponds to a negative $\log_{10}$ pathway adjusted *p*-value of 0.05.

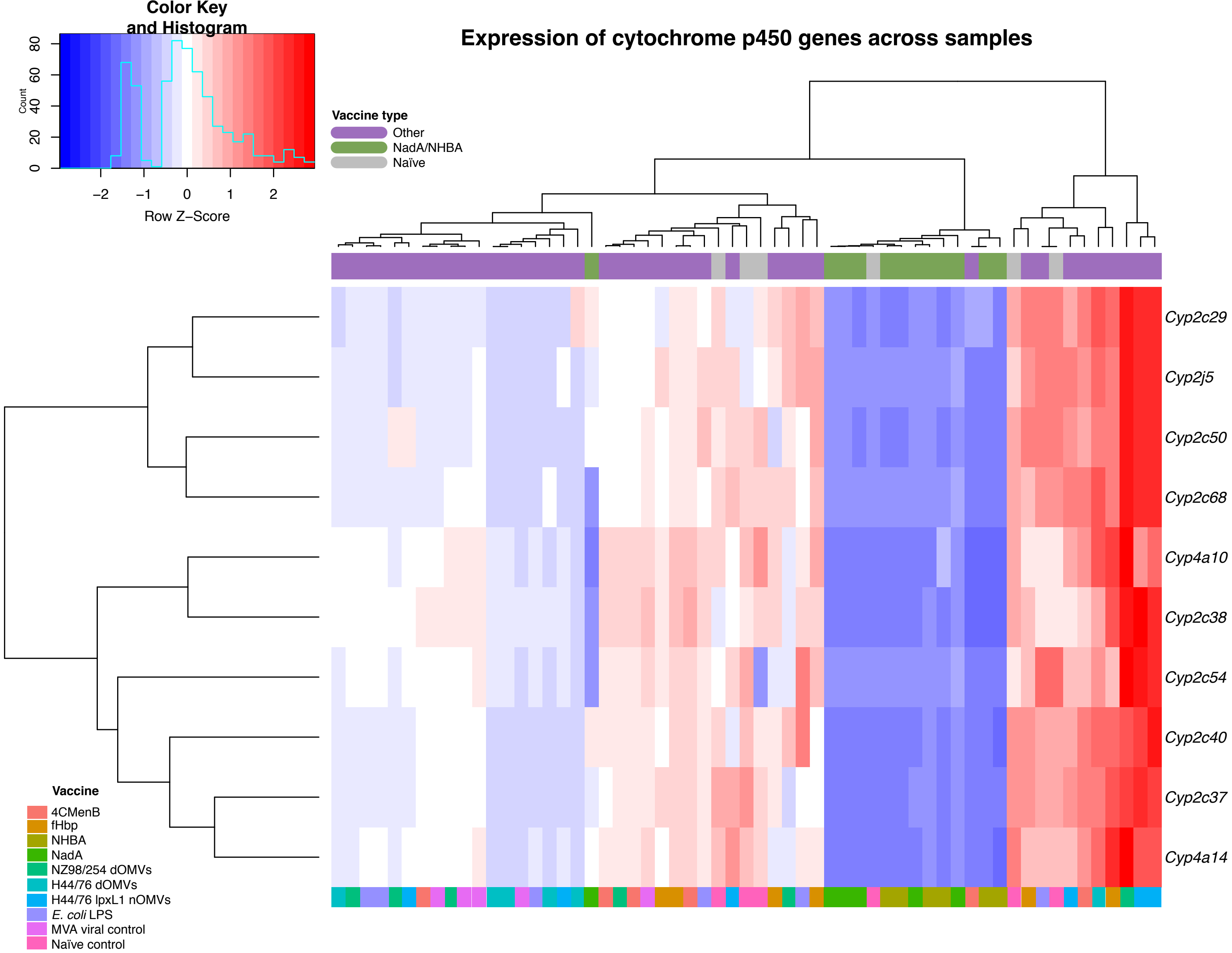

Supplementary Fig. 9. Hierarchical clustering of cytochrome P450 genes. Clustering across all samples of cytochrome P450 family of genes, a pathway identified as significantly downregulated in the NHBA and NadA groups.

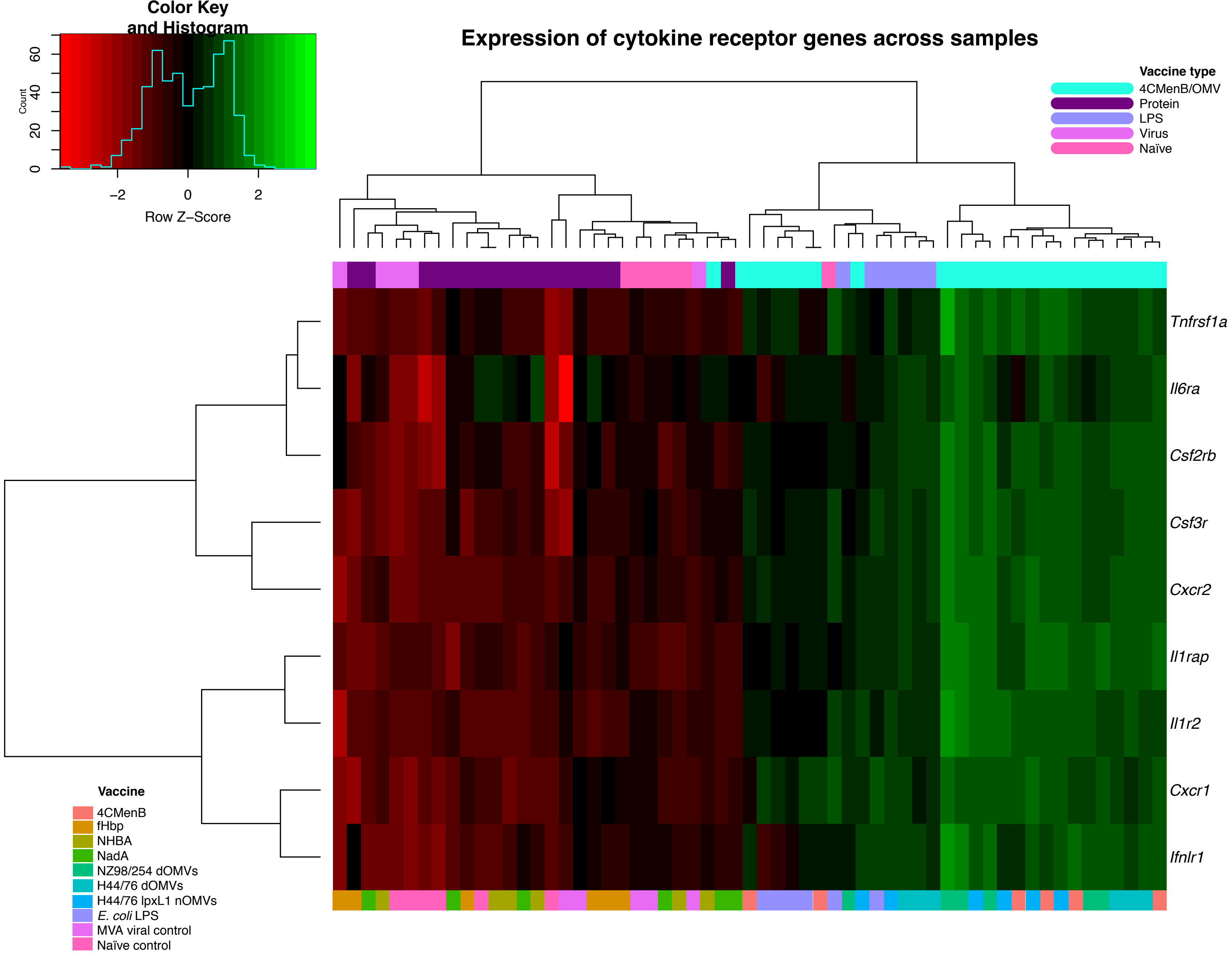

Supplementary Fig. 10. Hierarchical clustering of cytokine/chemokine receptors. Clustering across all samples of cytokine/chemokine receptor genes identified among the top significantly differentially expressed genes in the 4CMenB, OMV, and LPS groups.

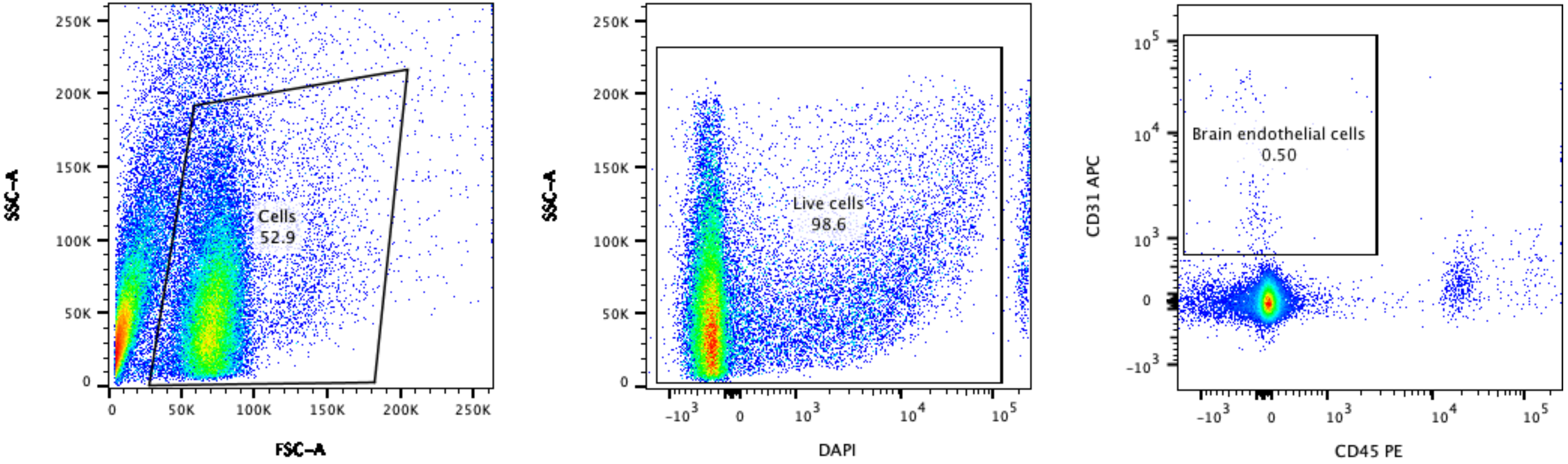

Supplementary Fig. 11. Gating strategy for brain endothelial cell isolation. Brain endothelial cells were identified as CD31+CD45- live cells.

Target gene	Primer
Il1r1	Mm00434237_m1
Il1rap	Mm00492638_m1
Il6ra	Mm00439653_m1
Tnfrsf1a	Mm00441883_g1
Tnfrsf1b	Mm00441889_m1
Ptges	Mm00452105_m1
Ptgs2	Mm00478374_m1
Pgk1	Mm01225301_m1

Supplementary Table 1. Primers used for real-time quantitative PCR of brain endothelial cells.

A Preamplification parameters

Step	Temperature (°C)	Time	Repeat
Enzyme activation	95	10 m	-
Denaturation	95	15 s	14 times
Anneal/extend	60	4 m	
Enzyme inactivation	99	10 m	-
	4		Hold

B RT-qPCR parameters

Step	Temperature (°C)	Time	Repeat
UNG incubation	50	2 m	-
Enzyme activation	95	10 m	-
Denaturation	95	1 s	40 times
Anneal/extend	60	20 s	

Supplementary Table 2. Thermocycling parameters. A. Parameters for preamplification of cDNA. **B.** Parameters for StepOnePlus™ real-time quantitative PCR.
